# Investigating the probiotic characteristics of four microbial strains with potential application in feed industry

**DOI:** 10.1371/journal.pone.0218922

**Published:** 2019-06-26

**Authors:** Jung-Ae Kim, Joel Bayo, Juncheol Cha, Yeon Jae Choi, Min Young Jung, Dae-Hyuk Kim, Yangseon Kim

**Affiliations:** 1 Center for Industrialization of Agricultural and Livestock Microorganisms, Jeongeup-si, Korea; 2 Department of Agricultural Convergence Technology, Chonbuk National University, Jeonju-si, Korea; 3 Chemport Inc. Naju-si, Korea; 4 International Agricultural Development and Cooperation Center, Chonbuk National University, Jeonju-si, Korea; 5 Department of Molecular Biology, Chonbuk National University, Jeonju-si, Korea; University of Huddersfield, UNITED KINGDOM

## Abstract

The present study aimed to evaluate the probiotic characteristics of certain microbial strains for potential use as feed additives. Three bacterial strains and a yeast previously isolated from different environments were investigated. The strains were subjected to molecular identification and established as *Lactobacillus paracasei* CP133, *Lactobacillus plantarum* CP134, *Bacillus subtilis* CP350 and *Saccharomyces cerevisiae* CP605. *Lactobacillus* sp. CP133 and CP134 exhibited antibiosis, antibiotic activity, and relative odor reduction ability. *Bacillus subtilis* CP350 was thermotolerant, reduced hydrogen sulfide gas and showed significant proteolytic activity, whereas *Saccharomyces cerevisiae* CP605 exhibited high acid and bile salt tolerance. In general, the isolates in this study demonstrated improved functional characteristics, particularly acid and bile tolerance and relative cell adhesion to HT-29 monolayer cell line. Results in this work provides multifunctional probiotic characteristics of the strains for potential development of probiotics and cleaning of the environment.

## Introduction

There is growing awareness of the health-promoting effects of probiotics for both humans and livestock, with particular relevance in the food, feed and pharmaceutical industries. Hotel and Cordoba [1] define a probiotic as a culture of living microorganisms that confers a health benefit to the host when administered in an adequate quantity. Probiotic microorganisms have been associated with immunomodulation, digestion and nutrient utilization efficiency (prebiotics), production of short-chain fatty acids and essential vitamins, and competitive exclusion of pathogenic microorganisms [2–4].

In addition, some probiotic organisms produce bacteriocins and metabolites such as lactic acid, which are harmful to pathogenic microorganisms, highlighting their ability to modulate the gut microbiota. Hence, bacteria that produce lactic acid as a major end product of carbohydrate fermentation are generally recognized as safe (GRAS) [5–7]. The probiotic *Lactobacillus* species such as *L*. *acidophilus*, *L*. *rhamnosus*, *L*. *gasseri*, *L*. *fermentum*, *L*. *plantarum* act as important probiotic since their strain-specific properties that are beneficial to health [8]. Especially *L*. *paracasei* and *L*. *plantarum* utilized as food supplementations for its high tolerance to acidic stomach condition and bile salt secretions in human and animals. *L*. *paracasei* and *L*. *plantarum* also have been applied in medical treatment of various chronic and cardiovascular diseases such as Alzheimer’s, Parkinson’s, diabetes, obesity, cancer, hypertension, urinogenital complication, liver disorders, etc. [9–11]. Furthermore, probiotic character and metabolites of *Bacillus* sp. make good resource of *Bacillus* in biotechnology area and food industry. Additionally, *Bacillus* sp. is highly resistant to heat and harsh gastric condition because of spore forming, promising them ideal as food additives for mammals [12].

To a great extent, bacteria belonging to the genera *Lactobacillus* and *Bifidobacterium*, neither of which include any major pathogenic species have been extensively recognized for their direct and associated benefits to the host [13]. Moreover, *Streptococcus* spp, *Lactococcus lactis*, *Escherichia coli*, *Enterococcus* spp, and *Saccharomyces boulardii* have been shown to confer similar health benefits in animals [13, 14]. Therefore, being naturally generic, the term ‘probiotics’ includes bacteria and yeast and their interactions, with beneficial manipulation of the gastrointestinal environment that improves the health of the host [15]. For example, when used as a biotherapeutic, *Saccharomyces cerevisiae* re-equilibrated the intestinal microflora, which demonstrates the efficacy of this yeast in treating chronic or recurrent diarrhea associated with *Clostridium difficile* [16].

To exhibit the direct and associated benefits, probiotic organisms must adapt to the host environment, for instance, survive passage down the gastrointestinal tract and eventually full establishment in the colon at sufficiently high numbers to ensure sustained benefit [17]. In the interest of conformity, the Food and Agricultural Organization (FAO) and World Health Organization (WHO) released a joint report on guidelines for the evaluation of probiotics [18], and subsequent revisions of the evaluation and selection criteria [19, 20] have defined prerequisite functions in identifying probiotic organisms.

To characterize the functional roles of probiotic organisms, attributes including adhesion and colonization of mucosal and epithelial cells [21], acid and bile salt tolerance [22, 23], proteolysis [24] and probiotic stability and vitality are considered vital. It is also important to note that the desirable effects of probiotics would be enhanced if the organisms have the ability to adhere, multiply and colonize the intestine, as adhesion is an important prerequisite for gastrointestinal colonization and facilitates the functional roles of probiotic organisms [25].

In the present study, we aimed to investigate isolates of certain bacterial strains and a yeast by identifying and determining their probiotic characteristics based on the gastrointestinal model, antibiosis, antibiotic sensitivity, intestinal cell adhesion, lysosomal and proteolytic activity and odor removal efficiency of nitrogenous odor compounds ammonia (NH_3_) and methylamine (CH_2_NH_2_) from swine slurry.

The results of this study may reveal the probiotic characteristics of the studied strains and substantially improve our understanding regarding the variety of probiotic micro-organisms known to have probiotic qualities and those classified as potential probiotic to be used as additives in the food and feed industries.

## Methods and materials

### Isolation of bacteria and yeast

Rice straw silage, Kimch, livestock slurry and farm soil were collected from South Jeolla province (Naju city, Korea), and processed for isolating new bacterial and yeast strains. These samples (1 g) were processed by crushing and suspending in physiological saline (10 mL) and then homogenizing. For enumeration, the 10 times dilution series of each homogenates were prepared using sterile saline solution and 0.1 mL samples were spread on MRS (de Man, Rogosa and Sharpe agar), LB (Luria-Bertani), and YPD (Yeast Potato Dextrose) agar plates, which were incubated at 37°C and 30°C for 24 to 48 hrs to obtain strains CP133, CP134, CP350, and CP605. As reference stains, *Saccharomyces cerevisiae* ATCC18824, *Bacillus subtilis* ATCC6051, and *Lactobacillus rhamnosus* GG ATCC53013 were obtained from Korean collection for Type Cultures.

### 16S rRNA and ITS gene amplification and sequencing

Genetic amplification and sequencing of the 16S rRNA and internal transcribed spacer (ITS) regions were performed. Briefly, the 16S rRNA gene of the bacterial strains isolated was amplified using the described universal primers 27F (5´-AGA GTT TGA TCC TGG CTC AG-3´) and 1492R (5´-GGT TAC CTT GTT ACG ACT T T-3´) [26]. For the yeast, ITS amplification was performed using primers ITS1 (5´-TCC GTA GGT GAA CCT GCG G-3´ and ITS4 (5´-TCC TCC GCT TAT TGA TAT GC-3´ [27]. The PCR reaction was performed with a high-fidelity polymerase (AccuPrime Taq DNA Polymerase System, Invitrogen) using manufacturers protocol and Biometra GmBH PCR machine (Germany). Sequencing of the amplicons for 16S rRNA was performed using the primers 785F (5'-GGA TTA GAT ACC CTG GTA-3') and 907R (5'-CCG TCA ATT CMT TTR AGT TT-3') and ITS1 (5'-TCC GTA GGT GAA CCT GCG G-3') and ITS4 (5'-TCC TCC GCT TAT TGA TAT GC-3') for fungi. The DNA sequences have been deposited in the NCBI GenBank DNA database under the accession numbers MK601692, MK601693, MK601694, MK602319 for CP133, CP134, CP350 and CP605 respectively.

### Phylogenetic analysis

Gene fragments were assembled using the SeqMan program (Lasergene software V7, DNASTAR, USA), Reference gene sequences were compared using BLAST [28] with gene sequences available in GenBank DNA databases (https://www.ncbi.nlm.nih.gov) and Ribosomal Database Project (RDP). Phylogenetic analysis of the 16S rRNA and ITS regions was performed using Molecular Evolutionary Genetic Analysis (MEGA) software, Version 7 [29]. Evolutionary relationships were constructed using the maximum likelihood method based on bootstrapping [30].

### Determination of probiotic characteristics of the bacterial strains and yeast

#### Acid and bile salt tolerance

Simulation of the gastrointestinal tract (GI) to evaluate the tolereance of the bacterial and yeast strains under low pH and high bile salt concentration in the present study was determined using a modified procedure [31]. For the study of tolerance to acidic condition with microbial strains, sterile PBS was adjusted to pH 2.5 (treatment) and 7.0 (control) using 1 M HCl. An overnight culture, 5 mL of the isolates (approximately 1x10^7^ CFU/mL) was incubated for 2 hrs at 30°C (CP350 and CP605) and 37°C (CP133 and CP134) with and without shaking, respectively. The bile tolerance of the strains was determined by growth in modified nutrient broth, YPD and MRS broth with 0.3% oxgall (Difco, USA) for 8 hrs using the same incubation temperature conditions described above for acid tolerance. After incubation, 10 times serial dilutions were spread on agar plates followed by 24 hrs of incubation at 30°C and 37°C. Acid and bile tolerance was evaluated by enumeration of viable colonies, and each assay was performed in triplicate. In both cases, survival was calculated using the formula.

$$\mathrm{Survivability}=\frac{Treatment\;CFU/ml}{Control\;CFU/ml}\times 100$$

#### Thermotolerance

The heat tolerance of the isolated bacterial and yeast strains was examined using a modified procedure [32–36]. Cultured cells grown overnight were suspended in sterile broth (YPD, MRS, and nutrient media) and 1mL of the culture cells (1–2 x 10^7^ CFU/mL) were subjected to 5 min, 10min, 20min and 30 min, 60 min of heat treatment at 30°C—80°°C—90°C—100°C, 37°C—50°C—60°C—70°C and 30°C—40°C—52°C in a heat block for *Bacillus* sp., *Lactobacillus* sp., and yeast strain, respectively; the initial treatment at 30°C and 37°C were considered as a control. Immediately after heat treatment, 10 times serial dilutions were prepared and spread on YPD, MRS and nutrient agar plates in triplicate. The plates were incubated at 30°C and 37°C for 24 hrs, and surviving cells were enumerated.

#### Antibiotic sensitivity

The sensitivity of the isolated microbial strains to a set of antibiotics was assessed using the E-test minimum inhibitory concentration (MIC) method (E-test bio Mẽrieux BIODISK, France) as previously described [37], with some modifications. Eleven antibiotic strips impregnated with a minimum inhibition concentration (MIC) range of 0.016–256 μg/ml of amoxicillin, ampicillin, clindamycin, gentamicin, kanamycin, metronidazole, tetracycline, vancomycin and erythromycin and 0.016–32 μg/ml of imipenem and trimethoprim-sulfamethoxazole against the target strains was employed. Fresh samples of target strains were inoculated onto agar plates including nutrient agar (CP350), YPD (CP605) and MRS (CP133 and CP134) (Difco, USA), and the E-test strips were laid on the agar; the plates were incubated at 30°C (NA and YPD plates) and 37°C (MRS plates) for 24 hrs. Antibiotic sensitivity was determined by reading the MIC as the antibiotic concentration at the point where dense colonial growth intersected the strip. Tests were performed in triplicate for each strain for optimization [38].

#### Antibacterial analysis

Strains were evaluated for antibacterial activities against economically important enteropathogenic microorganisms using a previously described disk diffusion method [39], with slight modifications. Five enteropathogenic bacteria were used as indicators of antibacterial activity: *E*. *coli* KCTC2617, *Salmonella* Derby NCCP12238, *Salmonella* Typhimurium NCCP10438, *Yersinia enterocolitica* NCCP11129, and *Yersinia pseudotuberculosis* NCCP11125. In brief, pathogenic strains were initially grown on appropriate media, with *E*. *coli* grown on Luria Bertani agar (LB), *Salmonella* spp. on Salmonella and Shigella agar (SSA) and *Yersinia* spp. on MacConkey agar at 30°C—37°C for 20 hrs. Diffusion disks of 8 mm in diameter were appropriately overlaid on the agar, and 1x10^6^ CFU/mL of the culture suspensions were dispensed onto the disks. The plates were incubated at 30°C—37°C for 24 hrs, and the diameters of the inhibition zone around each disk were measured.

#### Cell adhesion assay using intestinal epithelial cells

The ability of microbial cells to adhere to the intestinal lining was determined using HT-29 colonic carcinoma cells derived from the human small intestine, as adopted from a previous study [40], with slight modifications. Monolayers of HT-29 cells were prepared in DMEM medium (Sigma, USA) supplemented with 10% fetal bovine solution (FBS) (Sigma, USA) in 24-well tissue plates (BD Biosciences, San Jose, CA USA) at a concentration of 1 x 10^5^ cells/well. The cells were incubated at 37°C for 2 hrs together with 2 x 10^7^ CFU/mL of a cultured strain to test for adhesion. After incubation, the HT-29 cells were aspirated and washed three times with 1 X PBS to remove unbound microbial cells. Adherent cells were detached, and appropriate dilution series were prepared followed by enumeration of viable colonies on appropriate agar plates in triplicate.

#### Lysosomal activity test

Microbial resistance to lysosomal hydrolytic enzymes was evaluated using a fluorescent-based microplate reader according to the manufacturer's instructions (Enzchek Lysosome Assay Kit (E-22013), Molecular Probes, Leiden, Netherlands). A cell-free supernatant of the strains was obtained by centrifuging at 12,000 rpm for 3 mins. Enzyme activity was determined by incubating 25 μL of culture sample and assay buffer mixture in a 96-well fluorescein microplate reader equipped with standard fluorescein filters for 30 mins. Initial shaking of the mixture at 125 rpm on an orbital shaker for 5 sec was followed by 30 mins of reaction time. Quantitative lysosome activity was determined by measuring and comparing the fluorescence intensity detected using the optical density of 450 nm.

#### Proteolytic activity test

To assess the proteolytic activity of the bacterial and yeast strains, a previous method [41] using 2% skimmed milk, gelatin and fortified agar plates was used, with slight modifications. Briefly, paper discs (8 mm) were laid in the center of agar plates, and 50 μL of culture suspension (1–2 x 10^8^ CFU/mL) was dispensed onto the filter disc followed by incubation for 48hrs at 30°C (CP350 and CP605) and 37°C (CP133 and CP134). Protease enzyme activity was determined by measuring the clear zone formation around the paper discs. The tests were performed in triplicate for optimization purposes.

### Odor reduction potentiality

The odor reduction ability of the strains was evaluated using a modified method [42]. Slurry samples obtained from swine fed a basal diet were used in this analysis. Briefly, slurry samples were added to 1L container to occupy 70% of the container. Strains were added to the slurry to constitute 1% of the slurry. The container was fitted with a gas-tight lid with valves for gas measurement. The container was incubated at 35°C for 24 hrs to establish the gas production rate. The efficacy of odor reduction was determined by measuring the concentrations of ammonia and methylamine using a gas range detector (GV-100S, CA USA).

#### Statistical analysis

Statistical evaluation of the data was performed using analysis of variance with the general linear model for a randomized complete block design. All treatments were performed in triplicate, and Duncan’s multiple range test was applied to define mean differences between specific treatments. P<0.05 was considered to indicate a significant difference. All analyses were conducted using SAS software (version 9.1, 2004; SAS Institute. Inc., NC, USA).

## Results

### Identification of the bacterial strains and yeast

**Fig 1:**
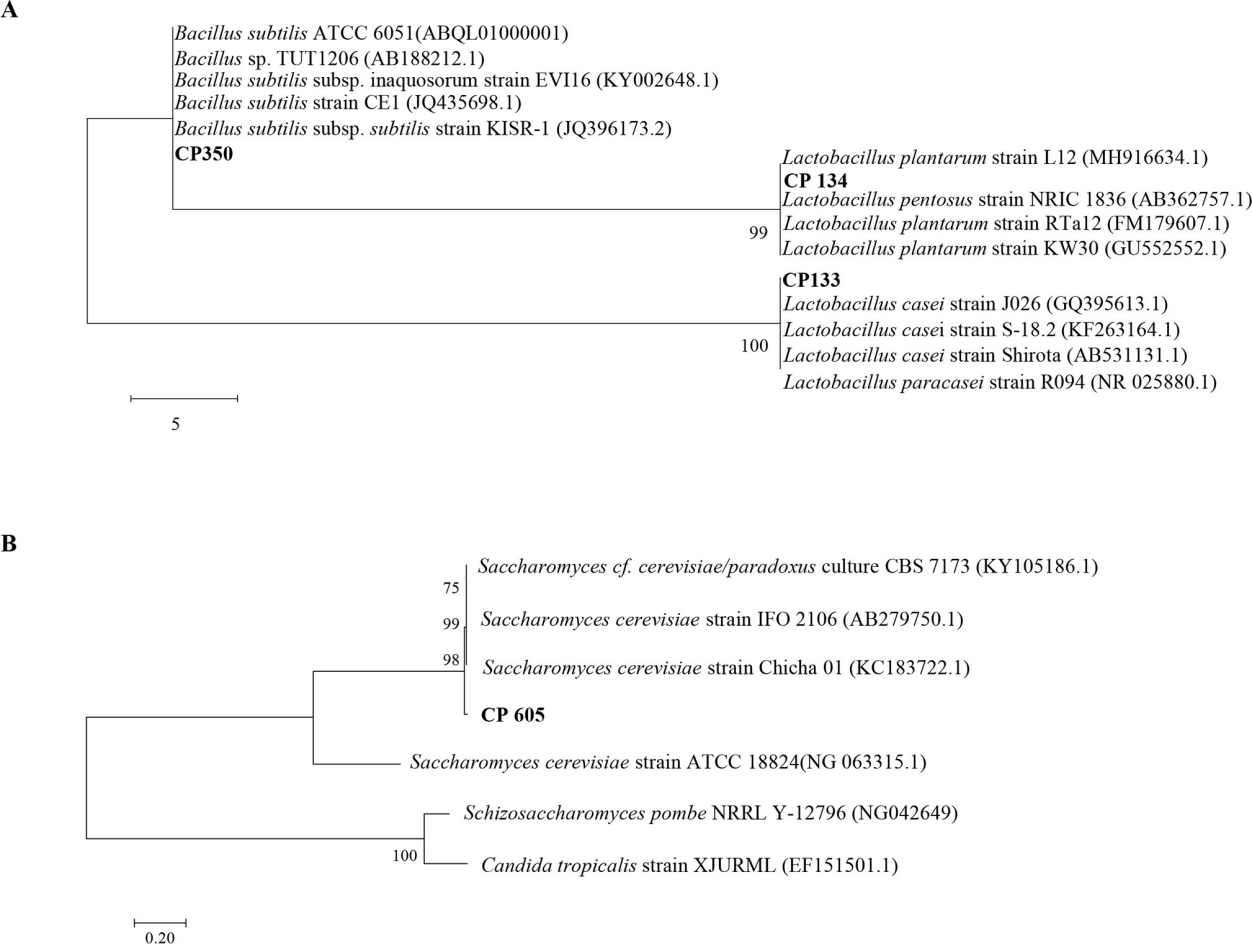
Molecular phylogenetic analysis showing the relationship of strains of CP133, CP134 and CP350 based on 16S rRNA **A.** and CP605 based on ITS **B.** using the maximum-likelihood method [32, 33]. Bootstrap values of 500 replicates are shown at the tree nodes, as generated using MEGA 7. The scale bar corresponds to 5 units (**A**) and 0.20 units (**B**) of the number of base substitutions per site.

The genomic DNA sequences of the strains were queried with available gene sequences in the GenBank library database. Strains CP133 form rice straw silage and CP134 from Kimchi are 99% homologous to *Lactobacillus paracasei* (NR_025880.1) and *Lactobacillus plantarum* (GU552552.1), respectively. The 16S rRNA genomic DNA sequence of CP350 isolated from livestock slurry exhibited a maximum identity of 100% with *Bacillus subtilis* subsp *subtilis* (JQ396173.2). In addition, the internal transcribed spacer sequences of CP605 isolated from farm soil show 99% similarity with *Saccharomyces cerevisiae* (KC183722.1). Furthermore, the phylogenic relationship between the bacterial strains was established based on maximum likelihood evolutionary distances of 16S rRNA sequences, as shown in Fig 1A. A similar evolutionary relationship for the yeast was determined based on its internal transcribed spacer gene sequence compared with type strains in GenBank (Fig 1B).

### Probiotic characteristics of the bacterial strains and yeast

#### Acid and bile tolerance, and intestinal adhesion activity

Acid and bile tolerance are essential properties of probiotic strains. It is suggested that a good probiotic must have the ability to withstand a pH of at least 3.0 because it is a common standard used to examine the acid tolerance of a probiotic culture. Evaluation of the probiotic characteristics based on gastrointestinal tract tolerance was determined at low pH and high bile salt. After 2 hrs incubation at pH 2.5, all strains exhibited stronger viability of greater than 97% (Table 1). CP134 showed significant (P<0.05) tolerance to 0.3% bile salts, with a tolerance rate of over 95% after 8 hrs of incubation. Although CP133 demonstrated remarkable acid tolerance, no viability of this strain was observed after 8 hrs of bile salt treatment. Conversely, CP605 was tolerant to both acid and bile, with over 97% tolerance. As positive control, *S*. *cerevisiae* ATCC18824, *B*. *subtilis* ATCC6051 and *L*. *rhamnosus* GG ATCC53103 were tested viability under condition at low pH and 0.3% bile salts.

**Table 1:** Acid and bile tolerance of the strains.

Variable	Condition	CP605	CP133	CP134	CP350	*S*. *cerevisiae* (ATCC18824)	*L*. *rhamnosus* GG (ATCC53103)	*B*. *subtilis* (ATCC6051)
Acid tolerance	2 hrs pH 7.0	7.18 ± 0.08^e^	9.21 ± 0.05^b^	8.46 ± 0.02^c^	6.02 ± 0.30^g^	7.55 ± 0.14^d^	9.50 ± 0.11^a^	6.45 ± 0.03^f^
	2 hrs pH 2.5	7.09 ± 0.07^c^	8.94 ± 0.12^a^	8.32 ± 0.05^b^	5.87 ± 0.03^e^	6.92 ± 0.08^d^	8.90 ± 0.01^a^	4.82 ± 0.05^f^
	Survivability (%)	**98.75**	**97.10**	**98.42**	**97.40**	**91.71**	**93.77**	**74.73**
Bile tolerance	8 hrs control	7.87 ± 0.09^f^	8.35 ± 0.04^c^	8.96 ± 0.05^b^	7.31 ± 0.09^g^	8.18 ± 0.00^d^	9.52 ± 0.05^a^	8.06 ± 0.03^e^
	8 hrs 0.3% Oxgall	7.70 ± 0.04^b^	0	8.57 ± 0.09^a^	5.67 ± 0.12^c^	2.22 ± 0.02^f^	2.95 ± 0.12^e^	5.51 ± 0.04^d^
	Survivability (%)	**97.94**	**0**	**95.65**	**77.66**	**27.17**	**30.99**	**68.31**

Data points represent means ± SD, and values within the same column with different superscript letters are significantly different at P<0.05. *The means are presented as log-transformed values of CFU/mL of the microbial strains*.

Table 2 presents the ability of the microbial cell to adhere to the intestinal lining using the human colonic carcinoma cell line HT-29. All strains displayed remarkable adherence to the HT-29 monolayer relative to the reference strain *S*. *cerevisiae* (81.17%), *B*. *subtilis* (82.90%), *and L*. *rhamnosus* GG (85.13%). Among the bacterial strains, CP133 and CP350 showed higher levels of cell adherence than did CP134 (Table 2). CP605 also adhered well to HT-29 cells (85.51%).

**Table 2:** Cell adhesion activity of the strains on the intestinal cell line.

Variable	Adhesion period	CP605	CP133	CP134	CP350	*S*. *cerevisiae* (ATCC18824)	*L*. *rhamnosus* GG (ATCC53103)	*B*. *subtilis* (ATCC6051)
Intestinal cell adhesion	0 hr	8.28 ± 0.03^c^	9.38 ± 0.00^a^	8.35 ± 0.02^b^	8.44 ± 0.00^b^	6.04 ± 0.07^e^	7.70 ± 0.05^d^	7.20 ± 0.10^d^
	2 hr	7.08 ± 0.01^a^	7.01 ± 0.01^a^	5.74 ± 0.03^e^	6.88 ± 0.03^c^	4.90 ± 0.05^f^	6.65 ± 0.08^b^	5.97 ± 0.02^d^
	Adhesion ability (%)	**85.51**	**74.66**	**68.71**	**81.61**	**81.17**	**85.13**	**82.90**

Cell adhesion is presented as the mean ± SD, and data followed by different superscript letters in the same column are significantly different (P<0.05).

#### Thermo-tolerance of the strains to increased temperature

**Fig 2:**
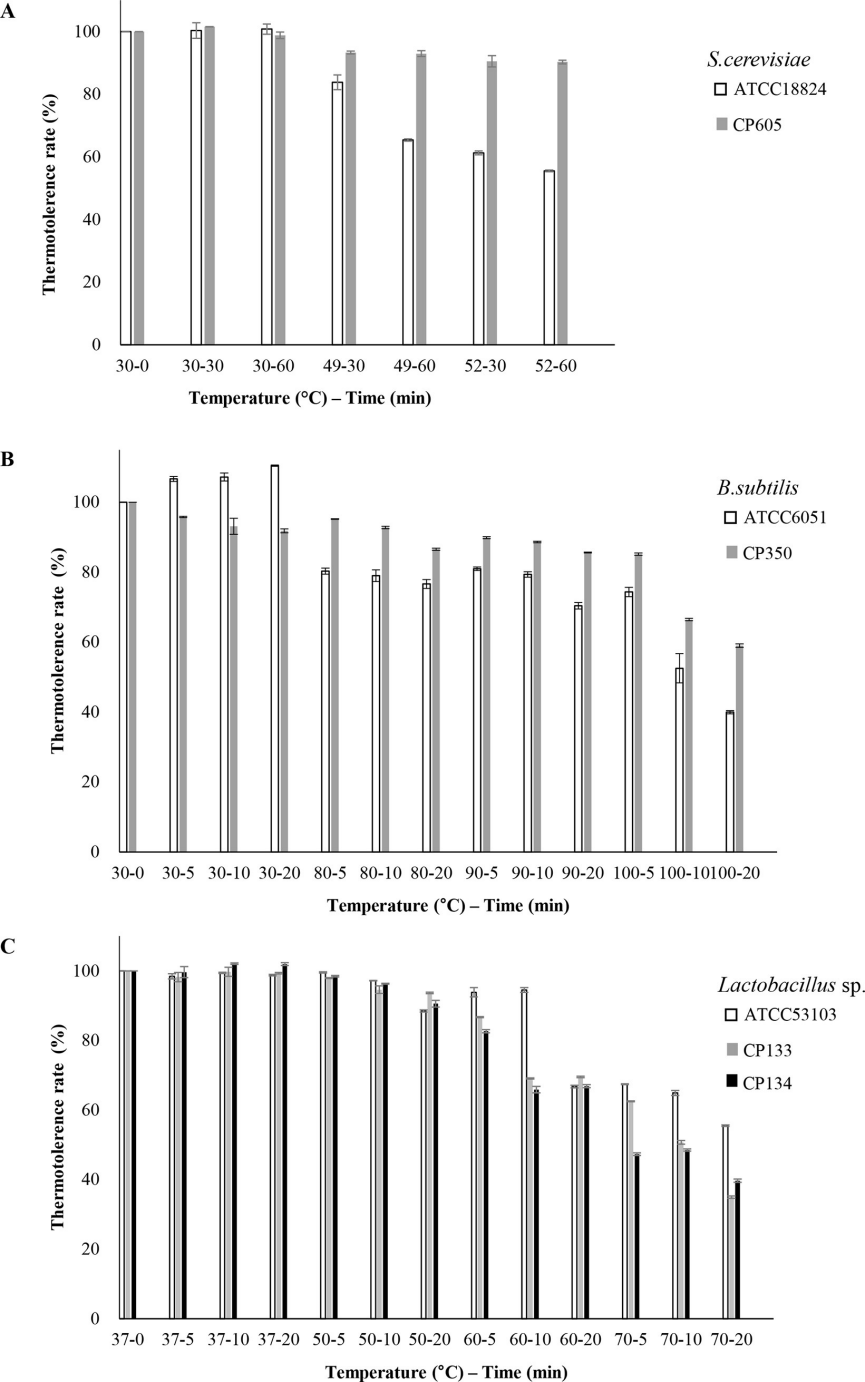
Thermotolerance to temperature upshifts of yeast stain CP605 A., *Bacillus* sp. CP350 B, *Lactobacillus* sp. CP133 and CP134 C. Data are expressed as means ± SD of the triplicate experimental set. Reference strains *S*. *cerevisiae* ATCC18824, *B*. *subtilis* ATCC6051 and *L*. *rhamnosus* GG ATCC53103 were tested as control.

Subjecting the strains to temperature increase resulted in a strong tolerance in viability (Fig 2). (Fig 2A and 2B) showed that the yeast strain CP605 and *Bacillus* sp. CP350 have consistent tolerance to temperature upshifts comparing to reference strains. *Lactobacillus* sp. CP133 and CP134 were tolerant to 60°C temperature increase, but showed general progressive loss to temperature increase (Fig 2C). Although, all the bacterial and yeast strains exhibited considerable tolerant to both of mammalian body temperature and industrial production condition.

#### Antibacterial activity and antibiotic sensitivity

The antibacterial activity of the strains against economically important enteropathogenic bacteria have summarized in Table 3. Interestingly, the strain CP133 and CP134 had good antibacterial activity against the indicator pathogenic strains tested. A clear zone of inhibition against *E*. *coli* KCTC2617 and *Y*. *pseudotuberculosis* NCCP11125 was found with CP134. Conversely, CP605 and CP350 were inactive against the indicator pathogens. Also the antibiotic sensitivity of the strains against commercially used antibiotics was also assessed using impregnated E-strips and shown in Table 4. CP605 exhibited absolute antibiotic resistance to all antibiotics. Whereas, CP133 was resistant to kanamycin, metronidazole, vancomycin, and trimethoprim; similarly, CP134 exhibited resistance to the same antibiotics, except for trimethoprim. In contrast, CP350 appeared to be resistant to only vancomycin and showed transient tolerance to amoxicillin and clindamycin.

**Table 3:** Antibacterial activity of the test strains against the indicator strains.

Test pathogenic strain	CP605	CP133	CP134	CP350
*Salmonella* Derby NCCP12238	-	+++	+++	-
*Salmonella* Typhimurium NCCP10438	-	++	++	-
*Yersinia enterocolitica* NCCP11129	-	+++	++	-
*Yersinia pseudotuberculosis* NCCP 11125	-	++	+++	-
*Escherichia coli* KCTC2617	-	+++	+++	-

The inhibition zone (mm) around the paper disc containing the microbial cell-free supernatant was classified as +++, >13 mm; ++, 10–12 mm; -, no inhibition zone.

**Table 4:** Minimum inhibitory concentrations of antibiotics against the test strains (μg/ml).

Antibiotic	Antibiotic sensitivity			
	CP605	CP133	CP134	CP350
Amoxicillin	R	≥1.0	≥0.19	**≥38**
Ampicillin	R	≥0.75	≥0.125	≥1.5
Clindamycin	R	≥0.023	≥0.032	**≥38**
Gentamicin	R	**≥64**	≥12	≥0.047
Imipenem	R	≥1.0	≥0.032	≥0.032
Kanamycin	R	R	R	≥0.094
Metronidazole	R	R	R	≥0.125
Tetracycline	R	≥0.50	≥12	≥0.064
Vancomycin	R	R	R	R
Erythromycin	R	≥0.094	≥0.50	≥3
Trimethoprim-sulfamethoxazole	R	R	≥2	≥0.047

Quantitative antibiotic sensitivity is expressed as the minimum inhibitory concentration against the microbial strains and classified as R, resistant (≥32 and 256 μg/ml) or presented as values in **bold** (weakly tolerant) and regular font (sensitive to the antibiotic).

#### Lysosomal and proteolytic activities

**Fig 3:**
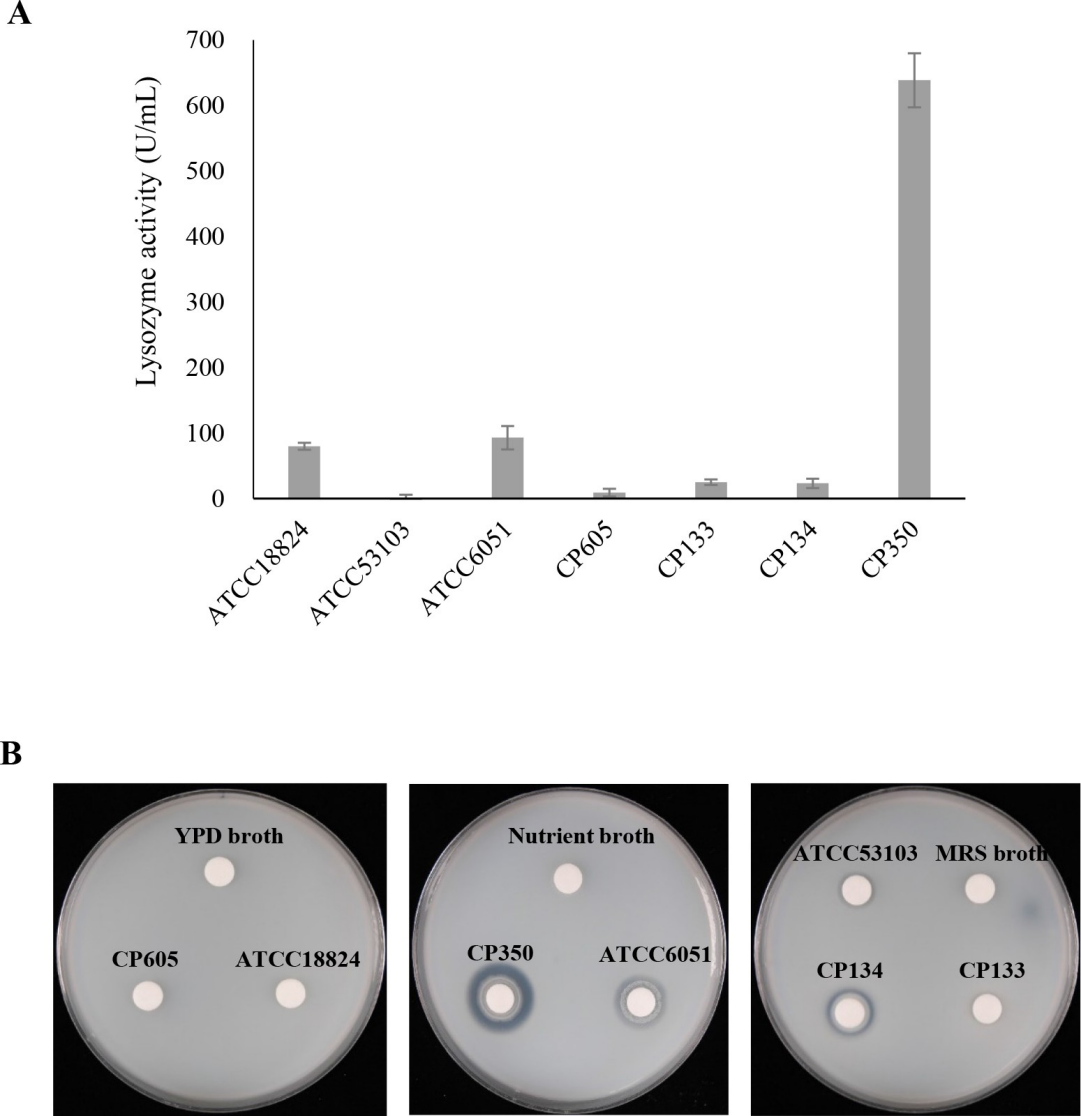
The antimicrobial effect of enzymes on the cell wall of *S*. *cerevisiae* CP605, *B*. *subtilis* subsp. *subtilis* CP350, *L*. *paracasei* CP133 and *L*. *plantarum* CP134. Evaluation of lysosomal hydrolytic enzymes of microbial strains **A.** Data are expressed as means ± SD. Statistical differences are marked with an asterisk (P<0.05). Degradation of the proteolytic enzyme substrate (casein) by protease secreted by microbial strains **B.** Reference strains *S*. *cerevisiae* ATCC18824, *B*. *subtilis* ATCC6051 and *L*. *rhamnosus* GG ATCC53103 were tested as control. YPD, MRS and nutrient media were tested as negative control for *S*. *cerevisiae*, *B*. *subtilis* and *Lactobacillus* sp. respectively.

The antimicrobial effect of lysosomes was evaluated, and the cell walls of CP605, CP133, and CP134 were significantly affected by lysosomal enzyme treatment and shown in Fig 3. The results also revealed that CP350 demonstrated marked resistance (P<0.05) to lysosomal activity, as shown by the high fluorescence intensity compared to the other strains (Fig 3A). Based on clear zone formation, CP350 and CP134 exhibited protease activity, whereas no clear zone was produced by CP133. Similarly, CP605 showed no protease activity (Fig 3B).

### Odor reduction potential by the bacterial strains and yeast

**Fig 4:**
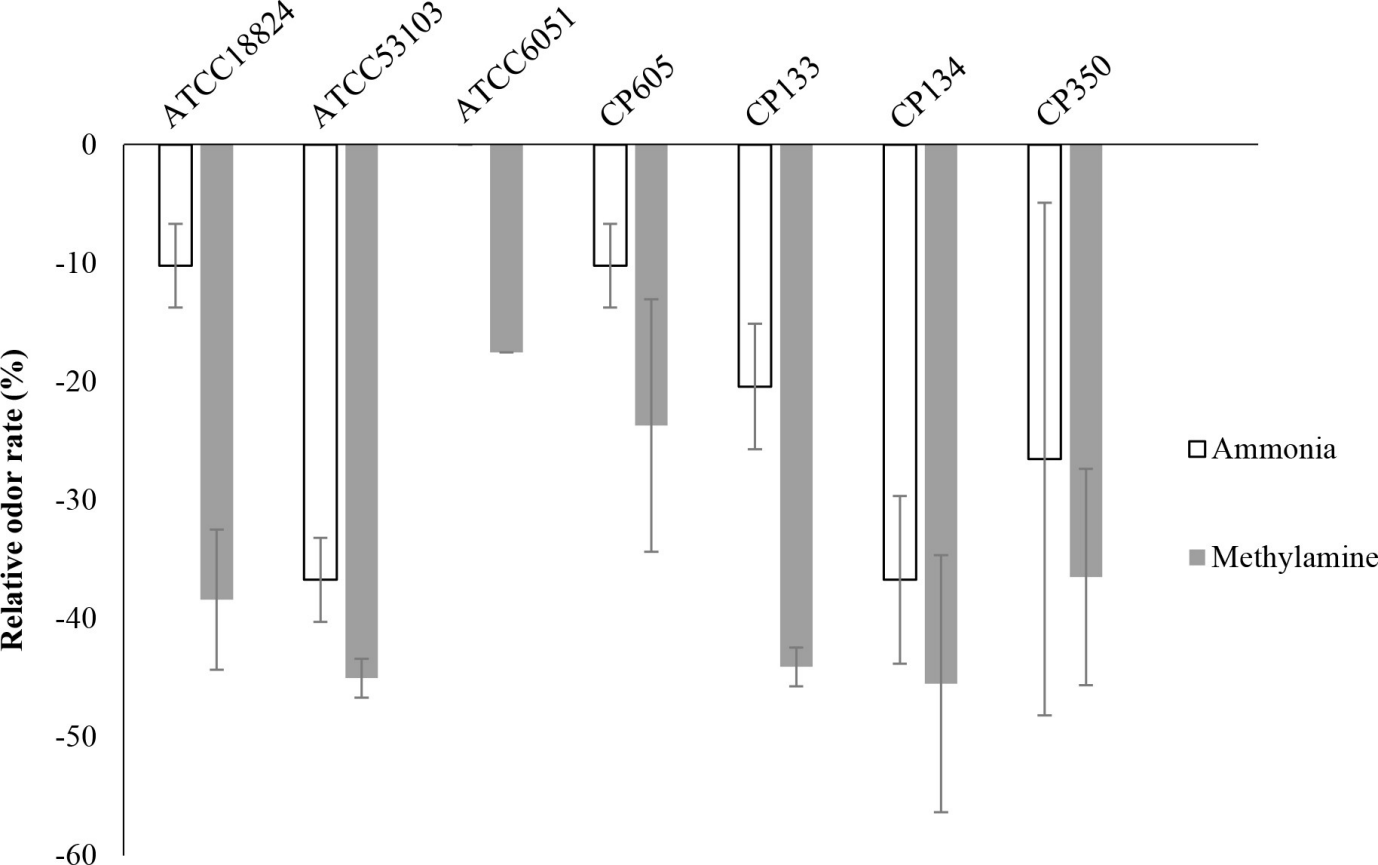
Odor reduction efficiency of *S*. *cerevisiae* CP605, *B*. *subtilis* subsp. *subtilis* CP350, *L*. *paracasei* CP133 and *L*. *plantarum* CP134. Data values represent the relative odor reduction rate of NH_3_, and CH_2_NH_2_ after 24 hrs of treatment of swine slurry with the strains. The results of reference strains *S*. *cerevisiae* ATCC18824, *B*. *subtilis* ATCC6051 and *L*. *rhamnosus* GG ATCC53103 were presented as control.

The odor reduction activity, e.g., ammonia, and methylamine in swine slurry, of the strains was evaluated after 24 hrs treatment. As shown in Fig 4, CP350 reduced ammonia and methylamine by 26.53% and 36.49%, respectively; CP133, CP134 decreased methylamine by 44.08%, 45.50%. However, the reference strains *S*. *cerevisiae* ATCC18824 highly reduced ammonia by 10.20% and methylamine by 38.39% compare to *S*. *cerevisiae* CP605. In general, a considerable reduction in indicator malodorous gasses, which are regarded as predominant odor compounds in swine slurries, was observed.

## Discussion

In principle, probiotic cells should exhibit characteristics of a large population able to survive and pass through the gastrointestinal tract with successful colonization of the intestinal epithelial cell lining for a considerable time period to confer associated health benefits to the host [43, 44]. Therefore, this study was designed to evaluate the several probiotic characteristics of four isolated bacterial strains and yeast based on several probiotic properties.

Previously *L*. *plantarum* FH185 showed high survivability at low pH and bile salt condition, as 97.4% and 96.6% respectively [45]. *Bacillus* sp. T3, T4, SM2 and JSP1 exhibited low cell viability at pH 2 below 65% but over 80% resistant to bile salt condition [46]. In the current study, all strains displayed high tolerance (P<0.05) to pH 2.5, over 97%. However, tolerance to bile salts varied from among the strains, with *S*. *cerevisiae* CP605 cells showing high bile salt tolerance. Although *Lactobacillus* sp. CP134 and *Bacillus subtilis* CP350 exhibited a high tolerance in bile salts, *Lactobacillus* sp. CP133 cells demonstrated no viability, suggesting that bile salt negatively impacted cell growth in this strain. Bile salts cause structural disorganization of the cell membrane, resulting in leakage of cell contents and ultimately death [47, 48], as demonstrated by the absolute death of cells of CP133 in 0.3% bile salt.

The thermotolerant behavior of the strains in Fig 2 revealed persistent tolerance to temperature upshifts by CP350 and CP605. Conversely, the tolerance rates of *Lactobacillus* sp. CP133 and CP134 declined with increasing temperature. Generally, the protective matrix in the microbial cell wall is destroyed at high temperature [49], suggesting low tolerance rates of *Lactobacillus* sp. CP133 and CP134. Further, *Bacillus subtilis* has been reported to synthesize heat shock proteins upon temperature increase [50], suggesting the thermo-tolerance for CP350. The isolates examined in this study, all were shown thermos-tolerance at 30°C and 37°C which is considered as the mammalian gastric environment and short generation time for industrial production condition.

Adhesion to the HT-29 monolayer differed significantly for *S*. *cerevisiae* CP605, *Lactobacillus* sp. CP133 and *Bacillus subtilis* CP350 (Table 2) compared with the reference strain *L*. *rhamnosus* GG. Moreover, *S*. *cerevisiae* strain in this study (CP605) demonstrated similar adhesion values to the recognized probiotic strain *L*. *rhamnosus* GG suggesting that it may be good *in vivo* colonizer. Mostly, adhesion to mucosal cells is important for the production of enzymes, lactic acid, vitamins and antimicrobial compounds. Interestingly, all strains in the current study indicated high adhesion to HT-29 monolayer cells compared to those of reference stains (Table 2). Nonetheless, factors such as pH, incubation time and cell concentration are critical in the ability of probiotic cells to adhere to the intestinal wall [51].

The antibacterial activity of *Lactobacillus* sp. CP133 and CP134 in the current study demonstrated a broad capacity to inhibit the growth of bacterial pathogens, which corresponds to previous findings of the antibacterial activity of *Lactobacillus* sp. isolated from fermentation conditions [52]. Inhibitory activity against the growth of pathogenic organisms is a known characteristic of lactic acid bacteria [53, 54], and the inhibitory ability of *Lactobacillus* sp. CP133 and CP134 can be associated with the production of bacteriocin-like metabolites, as previously reported [55]. Regardless, *B*. *subtilis* subsp. *subtilis* CP350 and *S*. *cerevisiae* CP605 in our study demonstrated no antibacterial activity against the tested pathogenic organisms.

*Lactobacillus* sp. shows resistance to glycopeptides, aminoglycosides and quinolone antibiotics [56, 57]. In the present study, *L*. *paracasei* CP133 and *L*. *plantarum* CP134 were found to be resistant to kanamycin, metronidazole, and vancomycin, which is consistent with previous studies [58, 59]. In addition, *Lactobacillus* sp. CP133 exhibited resistance to trimethoprim and a transient tolerance to gentamycin, whereas *B*. *subtilis* subsp. *subtilis* CP350 was resistant to amoxicillin and clindamycin. The other antibiotics (Table 4) tested inhibited the growth of the bacterial strains. Cell growth inhibition is usually associated with suppressing protein synthesis in target strain cells [59].

The resistance of the cell wall of probiotic strains to the antimicrobial hydrolytic enzymes contained in lysosomes is a vital property to protect against degradation [60]. Among the strains, *Bacillus subtilis* subsp. *subtilis* CP350 exhibited profound resistance to lysosomal activity (Fig 3A). It has been reported that resistance to cell wall degradation by lysosomal hydrolytic enzymes in *Bacillus* spp. is attributable to the inherent capacity of these cells to form endospores [61]. In addition, cell wall resistance to degradation by lysosomal enzymes occurs if the functional groups of the cell wall remain in the *N*-acetylation state, whereas conversion to *O*-acetylation facilitates degradation [62, 63]. The proteases synthesized by microbial strains are essential for protein metabolism and cell survival as well as identification [64, 65]. As was expected, *B*. *subtilis* subsp. *subtilis* CP350 showed high proteolytic activity (Fig 3B). Because they are the main producers of proteases, particularly alkaline proteases, proteolysis has been used as a means to identity bacteria of the genus *Bacillus* [66, 67].

In this study, besides probiotics characterization, odor reductions were examined with microbial strains CP605, CP350, CP133 and CP134. Odor which grows during the decomposition of swine waste makes complaints from the neighborhood and develops social issue. Recently bio-additives and microbial product have been applied to reduce the livestock odor [68]. Nitrogenous and short-chain fatty acids are generated during decomposition due to insufficient oxygen and high temperature [69, 70]. The odor reduction efficiency of the microbial strains was evaluated based on concentrations after 24 hrs of treatment. The results range from 10.2% to 45.5% for all bacterial strains, which was below the reduction rates compare to those of reference strains. These results promise these microbial strains to be strong candidates to odor reduction industry.

Current industrial probiotic microbial strains are predominantly isolated from terrestrial environment. It has been reported that marine yeasts are phenotypically distinct for terrestrial yeasts and highly tolerant to growth inhibitors such as salt, acid, and temperature [71]. Marine microorganisms, especially marine yeasts, have shown interesting probiotic properties [72, 73]. Marine yeasts, *S*. *cerevisiae* showed highly tolerant to salt revealing enhancement of glucose utilization for higher fermentation ability [74]. As the use of probiotics in aquaculture is becoming popular, probiotic characteristic of bacteria isolated from marine have been investigated. *L*. *plantarum* isolated from fish intestine enhanced immune system of tilapia leading the resistance to pathogen and bacteriocinogenic bacteria isolated from marine species were studied for probiotics characteristics for application in feed industry [75, 76].

In conclusion, the results obtained in this study reveal that *S*. *cerevisiae* CP605 isolated from farm soil, *B*. *subtilis* subsp. *subtilis* CP350 obtained from livestock slurry revealed well resistance to temperature, acidic and bile salt stresses, which is consider an important characteristic for probiotics. *L*. *plantarum* CP134 isolated Kimchi exhibited quality of highly adaptable in harsh gastric condition. Animal experiments are planned for future research to evaluate the impact on the animal’s health and productivity after application of all the isolates. Additionally, all the isolates show the considerable reduction rate in odor compounds of swine waste. Taken together, the results indicated that functional probiotic characteristics and capability in odor reduction are strain specific. Therefore, combination of these microbial strains may provide as strong resources for probiotics and odor reduction industries.
